# Supplementary material for: Nanoscale Zero-Valent Iron and Chitosan Functionalized Eichhornia crassipes Biochar for Efficient Hexavalent Chromium Removal
Source: Int J Environ Res Public Health. 2019 Aug 22;16(17):3046. doi: 10.3390/ijerph16173046 (PMC6747384; doi:10.3390/ijerph16173046)
Supplement: Supplementary file 1 [file ijerph-16-03046-s001.pdf]

Supporting information for

Nanoscale zero-valent iron and chitosan functionalized *Eichhornia crassipes* biochar for efficient hexavalent chromium removal

Xue-Li Chen [1], Feng Li [1,2,*], Xiao Jie Xie [1], Zhi Li [3] and Long Chen [2,*]

[1] School of Civil Engineering & Transportation, South China University of Technology, Guangzhou, 510640, China Emails: xueli_chen@hubu.edu.cn (X.L.C.), hjlifeng@scut.edu.cn (F.L.), 544949581@qq.com (X.J.X.)

[2] Department of Civil and Environmental Engineering, Northeastern University, Boston, MA 02115, USA Email: lo.chen@northeastern.edu (L.C.)

[3] California State University, San Bernardino, San Bernardino, CA 92407, USA Email: zlriverside2014@gmail.com (Z.L.)

* Correspondence: hjlifeng@scut.edu.cn (F.L.), lo.chen@northeastern.edu (L.C.)

Table S1. Statistical table of experimental variables and invariants.

<table>
<tr><td rowspan="2">pH</td><td>Invariant</td><td colspan="9">30 ℃, 30 min, 0.01mol/L NaNO3, 100.0mg/L Cr(VI)</td></tr>
<tr><td>Variable</td><td>2.0</td><td colspan="2">3.0</td><td>4.0</td><td>5.0</td><td colspan="2">6.0</td><td>7.0</td><td>8.0</td></tr>
<tr><td rowspan="2">t (min)</td><td>Invariant</td><td colspan="9">pH=2.0, 30 ℃, 0.01mol/L NaNO3, 100.0mg/L Cr(VI)</td></tr>
<tr><td>Variable</td><td>0</td><td>10</td><td>30</td><td>60</td><td>120</td><td>240</td><td>720</td><td>1080</td><td>1440</td></tr>
<tr><td rowspan="2">$NaNO_3$ (mg/L)</td><td>Invariant</td><td colspan="9">pH=2.0, 30 ℃, 30 min, 100.0mg/L Cr(VI)</td></tr>
<tr><td>Variable</td><td>0.0</td><td colspan="2">0.005</td><td colspan="2">0.05</td><td colspan="2">0.5</td><td colspan="2">5.0</td></tr>
</table>

Table S2. Parameters Characterizing the pore structure of the BC, nZVI-BC and C-nZVI-BC.

Sample	SA (m^2/g)	TPV (cm^3/g)	APR (nm)
C-nZVI-BC	833.1	0.61	4.43
nZVI-BC	748.99	0.59	4.85
BC	512.89	0.46	3.97

Table S3. Isotherm parameters obtained by experimental data for the sorption of Cr(VI) by BC, nZVI-BC and C-nZVI-BC.

<table>
<tr><td rowspan="2"></td><td rowspan="2">Q_e</td><td colspan="3">Langmuir</td><td colspan="3">Freundlich</td></tr>
<tr><td>Q_m</td><td>K_L</td><td>R^2</td><td>K_F</td><td>n</td><td>R^2</td></tr>
<tr><td>C-nZVI-BC</td><td>66.12</td><td>68.316</td><td>0.168</td><td>0.994</td><td>16.659</td><td>0.3</td><td>0.959</td></tr>
<tr><td>nZVI-BC</td><td>43.78</td><td>45.733</td><td>0.165</td><td>0.998</td><td>14.056</td><td>0.204</td><td>0.926</td></tr>
<tr><td>BC</td><td>14.62</td><td>27.171</td><td>0.179</td><td>0.99</td><td>4.392</td><td>0.277</td><td>0.987</td></tr>
</table>

Table S4. Kinetic parameters for the adsorption of Cr(VI) onto BC, nZVI-BC and C-nZVI-BC.

	Q_e	Pseudo-first-order			Pseudo-second-order			Elovich		
		q_e	K_1	R^2	q_e	K_2	R^2	a	b	R^2
C-nZVI-BC	52.304	54.184	0.016	0.939	56.8	2.939	0.947	9.658	0.324	0.882
nZVI-BC	34.029	35.023	0.020	0.941	36.408	1.748	0.956	5.589	0.659	0.840
BC	18.258	18.234	0.022	0.916	19.514	0.448	0.986	2.900	0.887	0.911

Table S5. The elemental composition and atom percent of C-nZVI-BC composite before and after Cr(VI) adsorption.

Element	Wt%		Atom percent	
	before	after	before	after
C	69.26	55.18	83.75	72.32
O	20.20	25.59	16.03	22.20
Si	0.11	0.07	0.05	0.04
Cr	-	8.96	-	2.39
Fe	10.43	10.20	3.17	3.05
Total	100.00		100.00	

Table S6. The quantitative XPS analysis of the C-nZVI-BC and nZVI-BC after Cr(VI) adsorption, Cr 2p.

	Cr(III)	Cr(VI)
nZVI-BC	74.74	25.26
C-nZVI-BC	59.95	40.05

Table S7. The maximal sorption capacity of biochars produced from different biomass feedstocks for Cr sorption from aqueous solutions.

Feedstock	Sorption capacity (mg g^{-1})	Reference
Sugar beet tailing	123	1
Coconut coir (250, 350, 500, 600 ℃)	31.1, 10.9, 7.9, 4.1	2
Peanut straw	25	3
Soybean straw	17.2	
Canola straw	14.6	
Rice straw	14	
Chlorella	18.86	4
Peanut hull	77.25	5
corn stack	20.04	6
sawdust	17.7	
wheat straw	14.39	
rice straw fed frass	32.59	7
sugarcane bagasse	43.122	8
corn straw	1.03	9
Rice straw-biochar colloids	10.4	10
Peanut hull	0.9	11
Mentha piperita	6.45	12
Artemisia argyi stem	161.92	13
Wheat straw	24.6	14
Wicker	23.6	
Rice husk	23.1	15
Cherry	16.01	16
Oleaster	24.65	
Eichhornia crassipes	20.6	This work

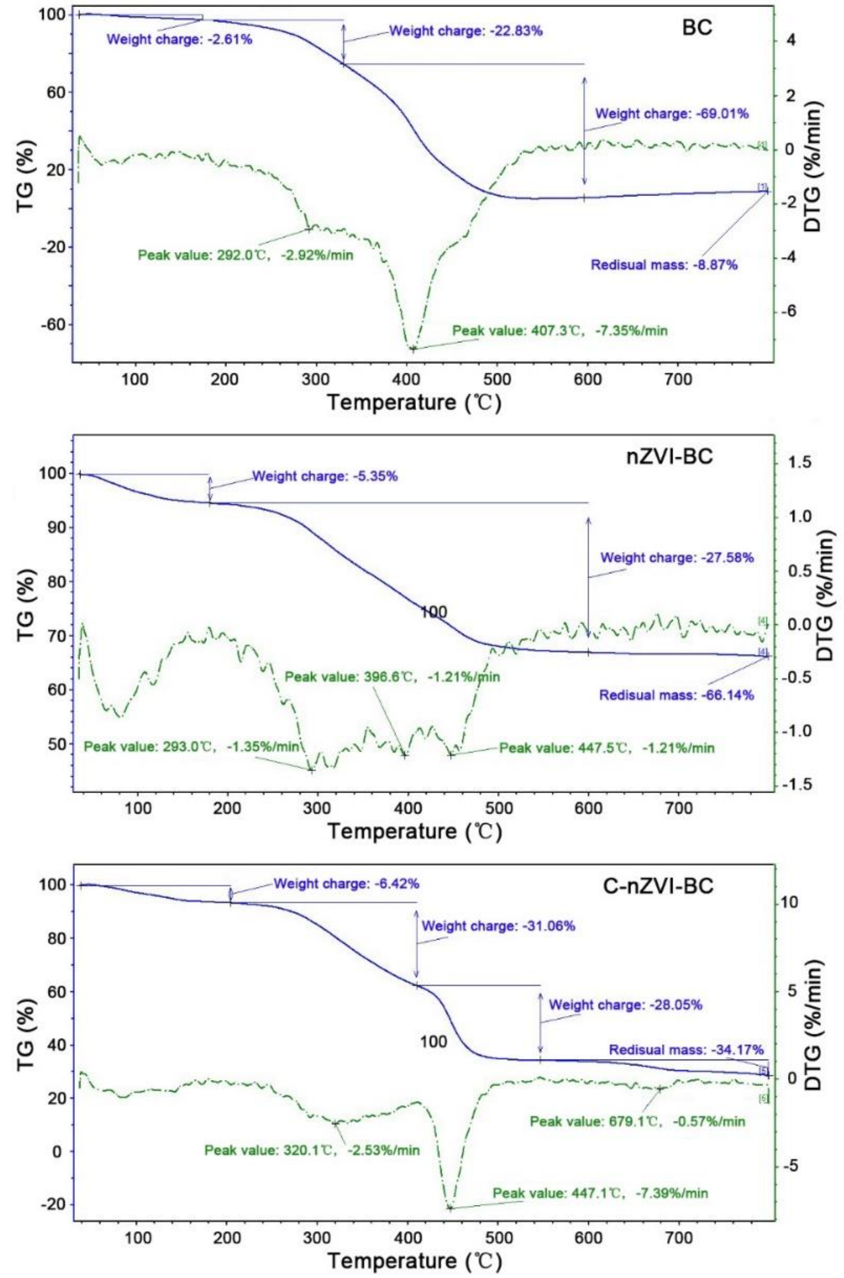

Figure S1. Thermogravimetric curve of BC, nZVI-BC and C-nZVI-BC.

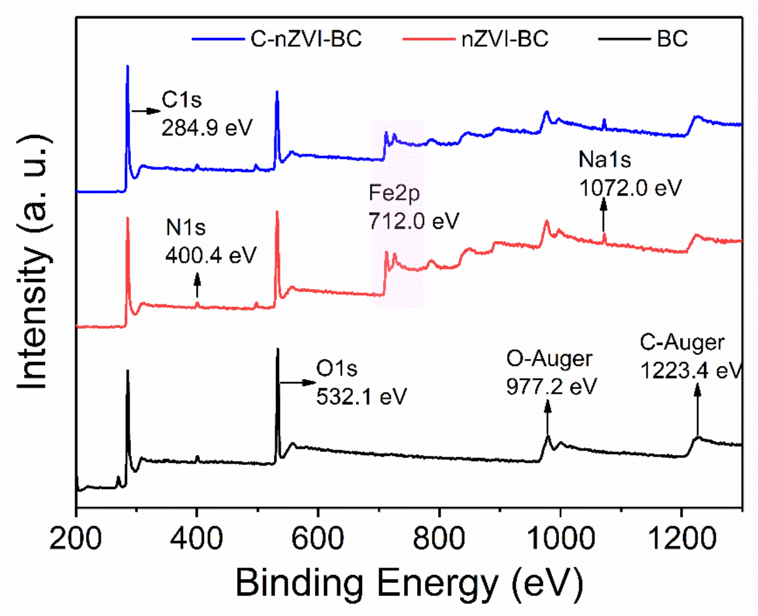

Figure S2. The XPS spectra of BC, nZVI-BC and C-nZVI-BC.

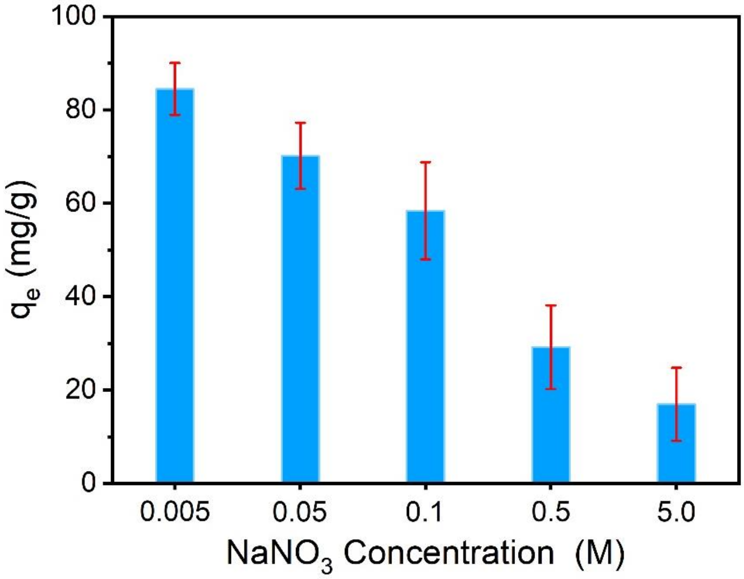

Figure S3. Effect of solubility of background solution (NaNO3) on the Cr(VI) adsorption by C-nZVI-BC. (Reaction conditions: Cr(VI) concentration c0 =100 mg/g; V = 100 mL; m = 100 mg; T = 30℃; pH = 2).

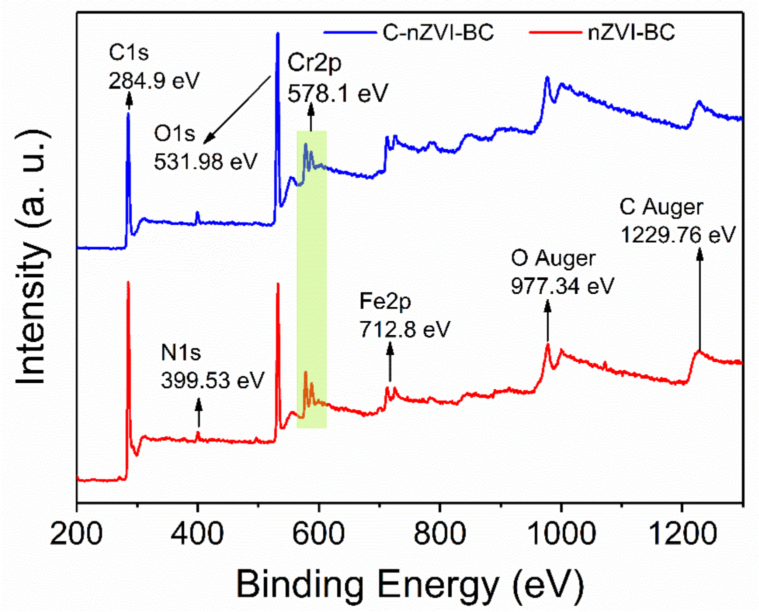

Figure S4. The XPS survey spectra of the nZVI-BC (red) and C-nZVI-BC(blue) after Cr-adsorption.

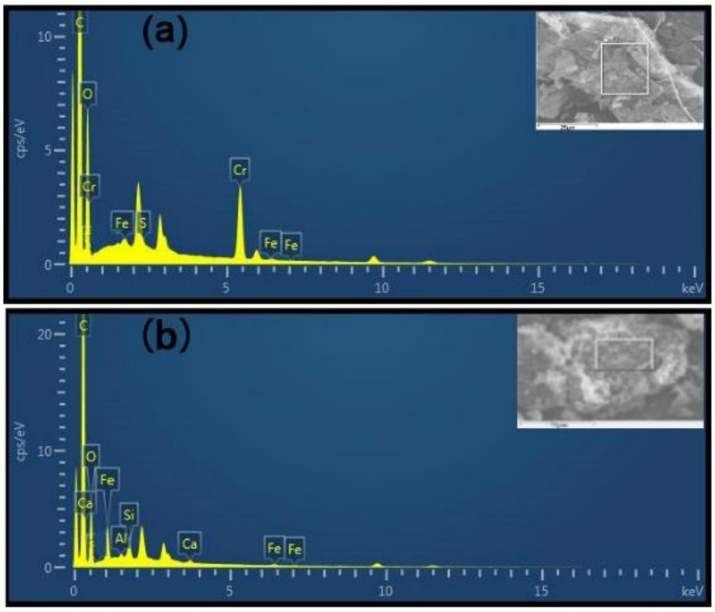

Figure S5. The EDX of C-nZVI-BC composite before (b) and after (a) Cr(VI) adsorption.

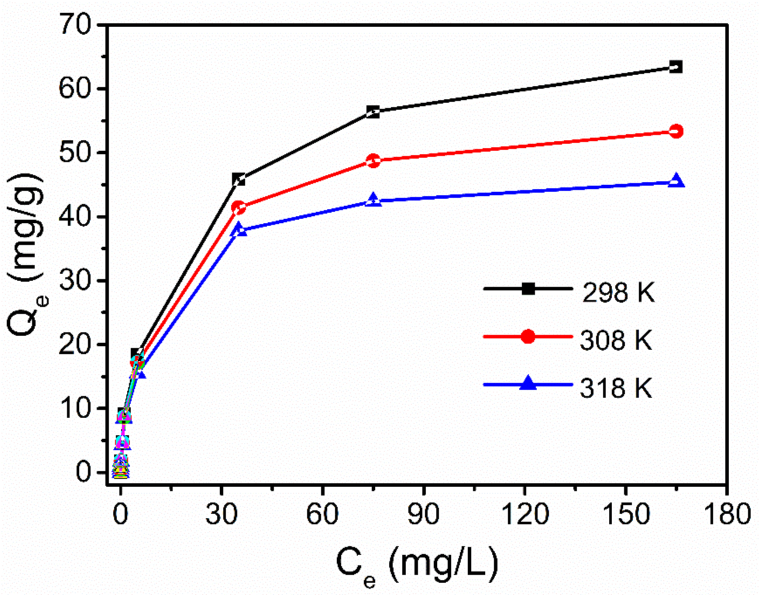

Figure S6. The Effect of temperature on the adsorption of Cr(VI) onto adsorbents.

Reference

1. Dong, X.L.; Ma, L.Q.; Li, Y. Characteristics and mechanisms of hexavalent chromium removal by biochar from sugar beet tailing. J. Hazard. Mater. 2011, 190, 909-915.
2. Shen, Y.S., Wang, S.L., Tzou, Y.M., Yan, Y.Y., Kuan, W.H. Removal of hexavalent Cr by coconut coir and derived chars e the effect of surface functionality. Bioresour. Technol. 2012, 104, 165-172.
3. Pan, J.J.; Jiang, J.; Xu, R.K. Sorption of Cr(III) from acidic solutions by crop straw derived biochars. J. Environ. Sci. 2013, 25, 1957-1965.
4. Amin, M.; Chetpattananondh, P. Biochar from extracted marine Chlorella sp. residue for high efficiency adsorption with ultrasonication to remove Cr(VI), Zn(II) and Ni(II). Bioresour. Technol. 2019, 289, 121578.
5. Han, Y.; Cao, X.; Ouyang, X.; Sohi, S.P.; Chen, J. Adsorption kinetics of magnetic biochar derived from peanut hull on removal of Cr (VI) from aqueous solution: effects of production conditions and particle size. Chemosphere 2016, 145, 336–341.
6. Peng, Z.Y.; Liu, X.M.; Chen, H.K.; Liu, Q.L.; Tang, J.C. Characterization of ultraviolet-modified biochar from different feedstocks for enhanced removal of hexavalent chromium from water. water sci. technol. 2019, 1705-1716.
7. Yang, S.S.; Chen, Y.D.; Zhang, Y.; Zhou, H.M.; Ji, X.Y.; He, L.; Xing, D.F.; Ren, N.Q.; Ho, S.H.; Wu, W.M. A novel clean production approach to utilize crop waste residues as co-diet for mealworm (Tenebrio molitor) biomass production with biochar as byproduct for heavy metal removal. Environ. Pollut. 2019, 252, 1142-1153.
8. Yi, Y.Q.; Tu, G.Q.; Zhao, D.Y.; Tsang, P.E.; Fang, Z.Q. Biomass waste components significantly influence the removal of Cr(VI) using magnetic biochar derived from four types of feedstocks and steel pickling waste liquor, Chem. Eng. J. 2019, 360, 212-220.
9. Liu, Y.Y.; Ma, S.Q.; Chen, J.W. A novel pyro-hydrochar via sequential carbonization of biomass waste: Preparation, characterization and adsorption capacity, J. Clean. Prod. 2018, 176, 187-195.
10. Khan N.; Clark I.; Sánchez-Monedero M.A. Physical and chemical properties of biochars cocomposted with biowastes and incubated with a chicken litter compost. Chemosphere 2016, 142, 14–23.

11. Banerjee S.; Mukherjee S.; Laminka-Ot A. Biosorptive uptake of Fe2+, Cu2+ and As5+ by activated biochar derived from Colocasia esculenta: isotherm, kinetics, thermodynamics, and cost estimation. J. Adv. Res. 2016, 7, 597–610.
12. Abhay Prakash Rawat; Singh, D.P. Synergistic action of adsorption and reductive properties of ash derived from distilled Mentha piperita plant waste in removal of Cr(VI) from aqueous solution. Ecotox. Environ. Safe. 2019, 176, 27–33
13. Song, J.Y.; He Q.L.; Hu X.L.; Zhang, W.; Wang, C.Y.; Chen, R.F.; Wang, H.Y. Ahmed Mosa Highly efficient removal of Cr(VI) and Cu(II) by biochar derived from Artemisia argyi stem, Environ. Sci. Pollut. Res. 2019, 26 (13), 13221-13234.
14. Tytłak A.; Oleszczuk P.; Dobrowolski R. Sorption and desorption of Cr(VI) ions from water by biochars in diferent environmental conditions. Environ. Sci. Pollut. Res. 2015, 22, 5985–5994.
15. Ma Y.; Liu W.J.; Zhang N.; Li Y.S.; Jiang H.; Sheng G.P. Polyethylenimine modifed biochar adsorbent for hexavalent chromium removal from the aqueous solution. Bioresour. Technol. 2014, 169, 403–408.
16. Kahraman H.T.; Pehlivan E. Cr6+ removal using oleaster (Elaeagnus) seed and cherry (Prunus avium) stone biochar. Powder Technol. 2016, 306, 61–67.
